# *P. aeruginosa* type III and type VI secretion systems modulate early response gene expression in type II pneumocytes in vitro

**DOI:** 10.1186/s12864-022-08554-0

**Published:** 2022-05-04

**Authors:** Emel Sen-Kilic, Annalisa B. Huckaby, F. Heath Damron, Mariette Barbier

**Affiliations:** 1 Department of Microbiology, Immunology, and Cell Biology, West Virginia University School of Medicine, Morgantown, WV USA; 2 Vaccine Development Center, West Virginia University Health Sciences Center, Morgantown, WV USA

**Keywords:** *P. aeruginosa*, Epithelial cells, Type III secretion system, Type VI secretion system, Early response genes, Transcriptomics, RNAseq

## Abstract

**Background:**

Lung airway epithelial cells are part of innate immunity and the frontline of defense against bacterial infections. During infection, airway epithelial cells secrete proinflammatory mediators that participate in the recruitment of immune cells. Virulence factors expressed by bacterial pathogens can alter epithelial cell gene expression and modulate this response. *Pseudomonas aeruginosa,* a Gram-negative opportunistic pathogen, expresses numerous virulence factors to facilitate establishment of infection and evade the host immune response. This study focused on identifying the role of two major *P. aeruginosa* virulence factors, type III (T3SS) and type VI (T6SS) secretion systems, on the early transcriptome response of airway epithelial cells in vitro.

**Results:**

We performed RNA-seq analysis of the transcriptome response of type II pneumocytes during infection with *P. aeruginosa *in vitro. We observed that *P. aeruginosa* differentially upregulates immediate-early response genes and transcription factors that induce proinflammatory responses in type II pneumocytes. *P. aeruginosa* infection of type II pneumocytes was characterized by up-regulation of proinflammatory networks, including MAPK, TNF, and IL-17 signaling pathways. We also identified early response genes and proinflammatory signaling pathways whose expression change in response to infection with *P. aeruginosa* T3SS and T6SS mutants in type II pneumocytes. We determined that T3SS and T6SS modulate the expression of *EGR1*, *FOS*, and numerous genes that are involved in proinflammatory responses in epithelial cells during infection. T3SS and T6SS were associated with two distinct transcriptomic signatures related to the activation of transcription factors such as AP1, STAT1, and SP1, and the secretion of pro-inflammatory cytokines such as IL-6 and IL-8.

**Conclusions:**

Taken together, transcriptomic analysis of epithelial cells indicates that the expression of immediate-early response genes quickly changes upon infection with *P. aeruginosa* and this response varies depending on bacterial viability and injectosomes. These data shed light on how *P. aeruginosa* modulates host epithelial transcriptome response during infection using T3SS and T6SS.

**Supplementary Information:**

The online version contains supplementary material available at 10.1186/s12864-022-08554-0.

## Background

*P. aeruginosa* is an opportunistic Gram-negative bacterium responsible for a wide array of infections in humans. If acute infections caused by *P. aeruginosa* are not treated, this bacterium can adapt to the lung environment, form biofilms, and persist, resulting in chronic infections that are difficult to eradicate [1]. Respiratory infections caused by this organism can be life-threatening in immunocompromised individuals and cystic fibrosis (CF) patients [2]. In particular, *P*. *aeruginosa* causes in CF patients both acute and chronic respiratory infections that negatively affect pulmonary function, morbidity, and mortality [3, 4]. This problem is worsened by the emergence of multidrug-resistant *P. aeruginosa* strains responsible for causing hard-to-treat infections, raising serious health concerns for susceptible individuals [5, 6]. Understanding the interactions of *P. aeruginosa* with its host can be beneficial for the development of novel therapeutics targeting bacterial virulence or host immune response mechanisms of bacterial clearance.

Airway epithelial cells constitute the first line of defense against pathogens during respiratory infections and form a physical barrier [7]. These cells participate in the innate immune response through mechanical mucociliary clearance and secretion of antimicrobial compounds in the airways [8, 9]. During infection, bacteria interact with airway epithelial cells, triggering a series of cellular host signaling pathways. Some of these signaling pathways lead to expression of immediate-early response genes (IEGs), which are controlled by constitutively active and/or post-translationally activated transcription factors [10, 11]. As a result, IEGs expression is induced rapidly after external stimulation [10]. Expression of IEGs shapes the host response by regulating the secondary response genes and downstream proinflammatory signaling cascades [11]. Infection with *P. aeruginosa* upregulates several IEGs such as *JUN*, *KLF2*, and *ZFP36* in epithelial cells [12, 13]. However, the molecular mechanisms involved in the induction of each of these IEGs during infection are unclear.

*P. aeruginosa* uses numerous strategies to establish infection and persist in the host. One of the primary virulence factors of *P. aeruginosa* is the type III secretion system (T3SS) that injects effector proteins directly into the cytoplasm [14]. In addition to T3SS, *P. aeruginosa* can also use a type VI secretion system (T6SS) to inject effector proteins inside eukaryotic cells [15]. This study aims to characterize the initial early host transcriptomic response produced by human airway epithelial cells to *P. aeruginosa,* and to elucidate the role of T3SS and T6SS in this process. To achieve this goal, transcriptomic analysis of type II pulmonary epithelial cells stimulated with live, heat-inactivated *P. aeruginosa,* and T3SS and T6SS mutants was performed. We showed that type II pneumocyte response to infection was characterized by up-regulation of pro-inflammatory genes, including genes whose products are involved in MAPK, TNF, and IL-17 signaling pathways. Upon stimulation with *P. aeruginosa,* most of the differentially upregulated transcription factors were immediate-early response genes known to be involved in proinflammatory responses. We identified that T3SS and T6SS affect *EGR1* and *FOS* genes expression in type II pneumocytes during infection. In addition, infections with T3SS and T6SS mutants were associated with two distinct host response profiles and transcription factor activation patterns including AP1, STAT1 and SP1, and lower levels of IL-6 and IL-8 secretion. Overall, this study is beneficial to understand the early activated transcriptomic signatures during *P. aeruginosa* infection and the role of injectosomes on the host response.

## Results

### ***P. aeruginosa*** triggers changes in expression of genes whose products are involved in proinflammatory pathways in lung epithelial cells

**Fig. 1:**
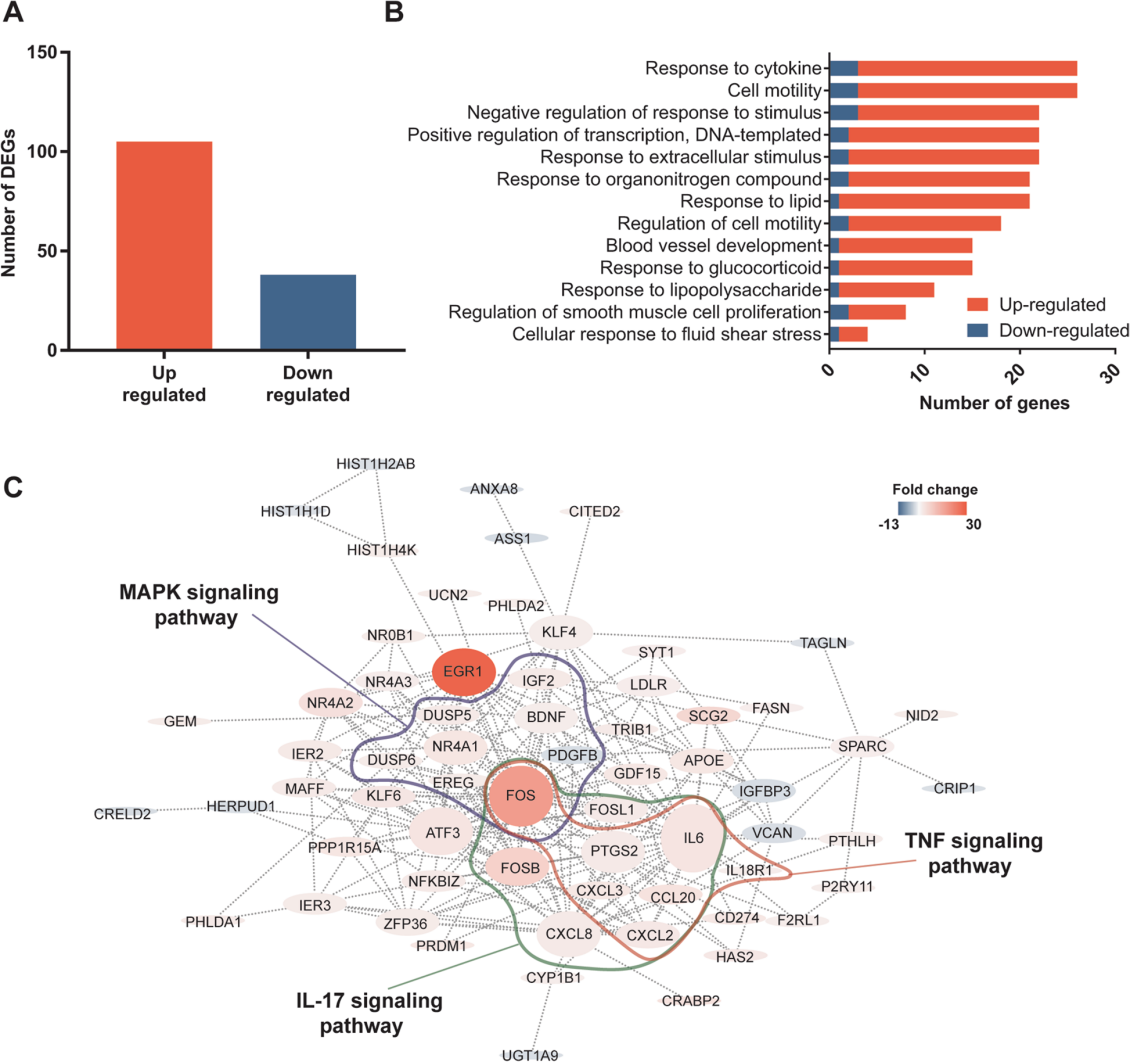
Characterization of epithelial cells transcriptomic response to *P. aeruginosa* at one-hour post-infection. **A** The number of differentially regulated genes in *P. aeruginosa* PA14 infected epithelial cells compared to non-infected control. Only genes with a *p*-value ≤ 0.05, fold change > 2, and average FPKM difference > 7 were included in the analysis. **B** GO term analysis of “biological processes” enriched in *P. aeruginosa* PA14 infected A549 cells compared to non-infected control. (*p* ≤ 0.05). **C** Protein-protein interaction network of differentially regulated genes in A549 cells during *P. aeruginosa* infection compared to non-infected control. The node size positively correlates with the degree of connectivity. The color of the nodes correlates with the fold change of the corresponding genes. Genes associated with KEGG “MAPK signaling pathway”, “IL-17 signaling pathway” and “TNF signaling pathway” are circled in blue, green and red, respectively. Only nodes with connection are shown

To gain insights into the response of lung epithelial cells to *P. aeruginosa* at the early stage of infection, transcriptomic responses of human type II pneumocytes (A549 cells) were analyzed after 1 h infection with *P. aeruginosa* PA14*.* The length of incubation was selected to identify early changes in gene expression [16, 17] without inducing cell death (Figure S1). The gene expression analysis was performed and genes with a *p-*value ≤ 0.05, fold change > 2, and average FPKM difference > 7 were further analyzed. *P. aeruginosa* infection led to changes in expression of 143 genes in epithelial cells compared to non-infected mock control. Among these genes, 73% (105 genes) were upregulated, and 27% were down-regulated (38 genes) compared to the non-infected mock control (Fig. 1A). The top 10 differentially upregulated and down-regulated protein-encoding genes are shown in Table 1.

**Table 1:** Top 10 differentially up- and down-regulated protein-encoding genes in epithelial cells post-*P. aeruginosa* infection

Gene Symbol	Official full name	Fold change	*P* value
*EGR1*	Early growth response 1	28.41	2.17 × 10^–24^
*FOS*	Fos proto-oncogene, AP-1 transcription factor subunit	17.47	9.93 × 10^–17^
*FOSB*	FosB proto-oncogene, AP-1 transcription factor subunit	7.39	1.05 × 10^–5^
*SCG2*	Secretogranin II	6.17	2.14 × 10^–4^
*SLC6A15*	Solute carrier family 6 member 15	4.59	1.01 × 10^–4^
*NR4A2*	Nuclear receptor subfamily 4 group A member 2	4.39	5.95 × 10^–7^
*CCL20*	C–C motif chemokine ligand 20	4.15	1.07 × 10^–4^
*CRABP2*	Cellular retinoic acid binding protein 2	4.10	8.12 × 10^–7^
*ATF3*	Activating transcription factor 3	3.51	1.02 × 10^–5^
*IL6*	Interleukin 6	3.50	3.65 × 10^–3^
*IGFBP3*	Insulin like growth factor binding protein 3	-2.27	4.71 × 10^–6^
*VCAN*	Versican	-2.30	4.91 × 10^–8^
*ANXA8*	Annexin A8	-2.32	3.62 × 10^–4^
*FICD*	FIC domain protein adenylyltransferase	-2.34	1.00 × 10^–2^
*CRELD2*	Cysteine rich with EGF like domains 2	-2.35	6.92 × 10^–3^
*TM4SF4*	Transmembrane 4 L six family member 4	-2.41	1.21 × 10^–4^
*ASS1*	Argininosuccinate synthase 1	-2.48	8.65 × 10^–3^
*FRMD3*	FERM domain containing 3	-2.65	3.74 × 10^–4^
*TC2N*	Tandem C2 domains, nuclear	-3.32	3.52 × 10^–5^
*FAM25C*	Family with sequence similarity 25 member C	-4.36	2.63 × 10^–5^

To understand the biological processes associated with the products of the genes differentially regulated during *P. aeruginosa* infection, GO term and KEGG pathway analysis was performed (Fig. 1B-C). In addition, a protein–protein interaction network with differentially expressed protein-encoding genes during *P. aeruginosa* infection was generated using STRING V11.0 [18] to determine what molecular networks are activated upon *P. aeruginosa* infection (Fig. 1C). We identified that molecular networks and pathways associated with pro-inflammatory responses and external stimuli are differentially regulated in epithelial cells upon infection with *P. aeruginosa* (Fig. 1B-C). In particular, protein-encoding genes associated with MAPK, TNF, and IL-17 signaling pathways were upregulated in response to *P. aeruginosa* in airway epithelial cells.

**Fig. 2:**
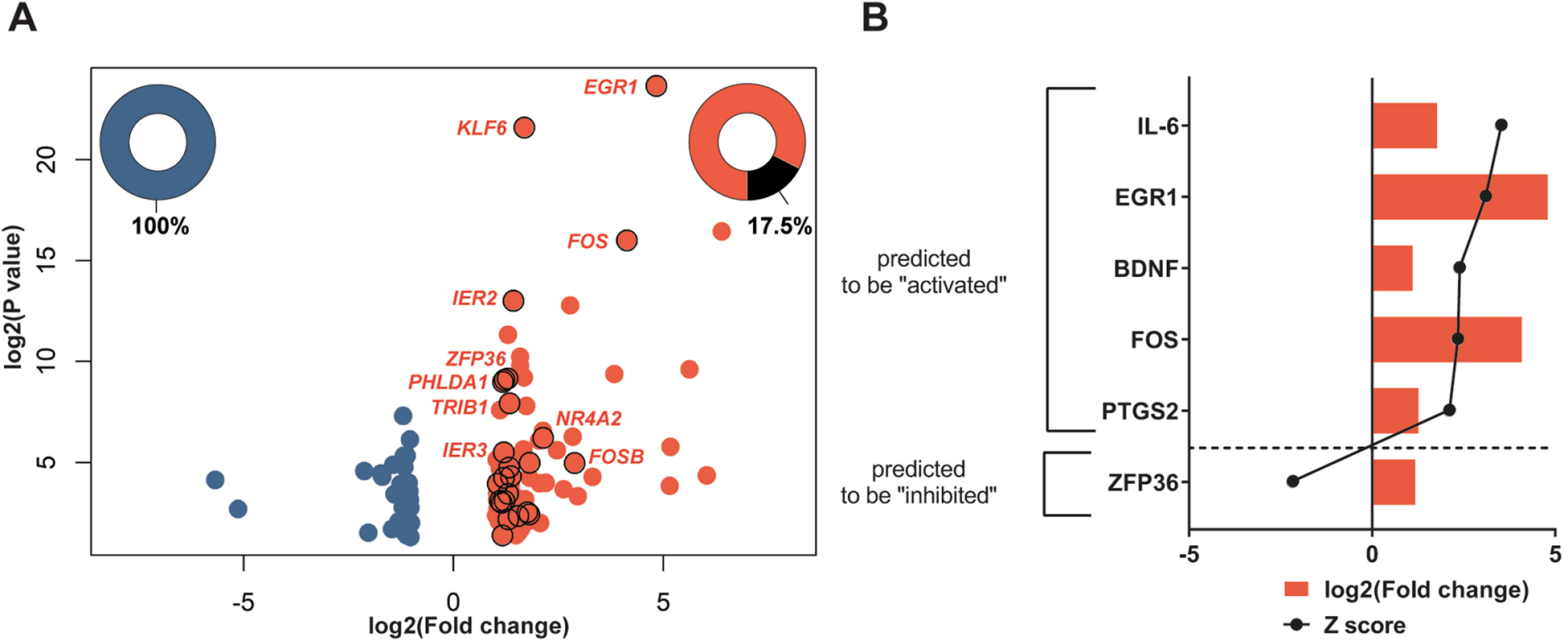
Immediate-early response genes in epithelial cells are upregulated upon *P. aeruginosa* infection. **A** Volcano plot of differentially expressed genes. Only genes with a *p*-value ≤ 0.05, fold change > 2, and average FPKM difference > 7 were analyzed as differentially regulated genes. Upregulated genes in response to PA14 compared to non-infected control are shown in red and down-regulated genes are shown in blue. Immediate-early response genes are outlined in black. Black proportion on the pie charts shows the percentage of immediate-early response genes in total differentially up or downregulated genes **B** Differentially regulated immediate-early response genes in *P. aeruginosa* PA14 infected epithelial cells with predicted upstream regulator function. Activation z-scores were calculated using IPA [24]. Only upstream transcriptional regulators with z-score > 2.0 or z-score < -2.0, and *p*-value < 0.01 are shown

Among the genes significantly upregulated in response to *P. aeruginosa* infection, 17.5% of them encoded transcription factors and proteins previously identified as IEGs (Fig. 2A, Table 2). The top 2 IEGs upregulated in epithelial cells after infection with *P. aeruginosa* were the genes encoding for the transcription factors EGR1 and c-Fos. The expression of these two genes was validated by qPCR (Figure S2). EGR1 and c-Fos are known to be involved in response to extracellular stimuli and play a role in regulating expression of genes with diverse functions including regulating inflammation, cell proliferation, and signal transduction [19–21]. In general, the products of IEGs play a major role in the activation of downstream signaling pathways essential for stimulating an immune response [11].

**Table 2:** Immediate-early response gene dataset

Gene symbol	Official full name	Fold change	*P* value	Refs
*EGR1*	Early growth response 1	28.41	2.17 × 10^–24^	[16, 17]
*FOS*	Fos proto-oncogene, AP-1 transcription factor subunit	17.47	9.93 × 10^–17^	[16, 17]
*FOSB*	FosB proto-oncogene, AP-1 transcription factor subunit	7.39	1.05 × 10^–5^	[16, 17]
*NR4A2*	Nuclear receptor subfamily 4 group A member 2	4.39	5.95 × 10^–7^	[16]
*ATF3*	Activating transcription factor 3	3.51	1.02 × 10^–5^	[16, 22]
*IL6*	Interleukin 6	3.50	3.66 × 10^–3^	[16, 22]
*NR4A1*	Nuclear receptor subfamily 4 group A member 1	3.38	2.92 × 10^–3^	[16]
*KLF6*	Kruppel like factor 6	3.22	2.50 × 10^–22^	[22, 23]
*GDF15*	Growth differentiation factor 15	2.92	4.31 × 10^–3^	[22]
*IER2*	Immediate early response 2	2.68	9.76 × 10^–14^	[17, 22]
*MAFF*	MAF bZIP transcription factor F	2.57	4.75 × 10^–5^	[22]
*TRIB1*	Tribbles pseudokinase 1	2.52	1.15 × 10^–8^	[17]
*DUSP5*	Dual specificity phosphatase 5	2.50	1.76 × 10^–5^	[16]
*FOSL1*	FOS like 1, AP-1 transcription factor subunit	2.47	6.45 × 10^–3^	[22, 24]
*PTGS2*	Prostaglandin-endoperoxide synthase 2	2.46	3.51 × 10^–4^	[22, 25, 26]
*PHLDA1*	Pleckstrin homology-like domain family A member 1	2.44	6.94 × 10^–10^	[27, 28]
*ZFP36*	ZFP36 ring finger protein	2.32	7.91 × 10^–10^	[16]
*DUSP6*	Dual specificity phosphatase 6	2.32	8.04 × 10^–4^	[16]
*EREG*	Epiregulin	2.30	5.69 × 10^–5^	[27, 29]
*GEM*	GTP binding protein overexpressed in skeletal muscle	2.29	3.09 × 10^–6^	[16]
*IER3*	Immediate early response 3	2.26	1.00 × 10^–9^	[16, 17]
*BDNF*	Brain derived neurotrophic factor	2.21	9.78 × 10^–4^	[22, 30]
*NR4A3*	Nuclear receptor subfamily 4 group A member 3	2.24	4.43 × 10^–2^	[16]
*KLF4*	Kruppel like factor 4	2.15	7.92 × 10^–4^	[23]
*LDLR*	Low density lipoprotein receptor	2.07	1.11 × 10^–4^	[16, 22]
*RHOB*	Ras homolog family member B	1.98	2.13 × 10^–2^	[31]
*KLF2*	Kruppel like factor 2	1.97	2.73 × 10^–2^	[22, 23]
*HES1*	Hes family bHLH transcription factor 1	1.82	6.19 × 10^–4^	[22]

The expression of IEGs is controlled by transcriptional regulators already present in the cell ready for signal-mediated activation such as phosphorylation. As a result, the induction of IEG expression happens rapidly upon stimulation and does not require de novo protein synthesis [16]. To identify the upstream regulators potentially involved in regulating gene expression in response to *P. aeruginosa*, Ingenuity Pathway Analysis (IPA) was performed [32]. The IPA upstream regulator analysis predicts the transcriptional regulators that are activated/inhibited based on the differential expression of downstream genes. One hundred eighty-four upstream transcriptional regulators were predicted to be activated or repressed in *P. aeruginosa* infected epithelial cells. Among these genes, six upstream regulators were also IEGs differentially regulated in response to *P. aeruginosa* infection (Fig. 2B). The IPA upstream regulator and Cytoscape network analyses predicted that transcription factors encoded by EGR1 and FOS were activated, acted as upstream regulators, and were important hub proteins in the protein interaction network (Fig. 2B, Fig. 1C). In addition, the gene encoding IL-6 had the highest positive activation z-score and IL-6 had the most interactions in the protein interaction network (Fig. 2B, Fig. 1C). Interestingly, the gene encoding ZFP36 was predicted to be inhibited even though it was measured as upregulated in epithelial cells after infection with *P. aeruginosa*, highlighting the importance of combining in silico prediction analyses with in vitro transcriptomic studies (Fig. 2B). Overall, these data show that during early type II pneumocyte infection with *P. aeruginosa*, IEGs are one of the main classes of genes upregulated, and products of these IEGs are predicted to participate in the regulation of gene expression.

### *P. aeruginosa* T3SS and T6SS trigger distinct changes in gene expression in type II pneumocytes

**Fig. 3:**
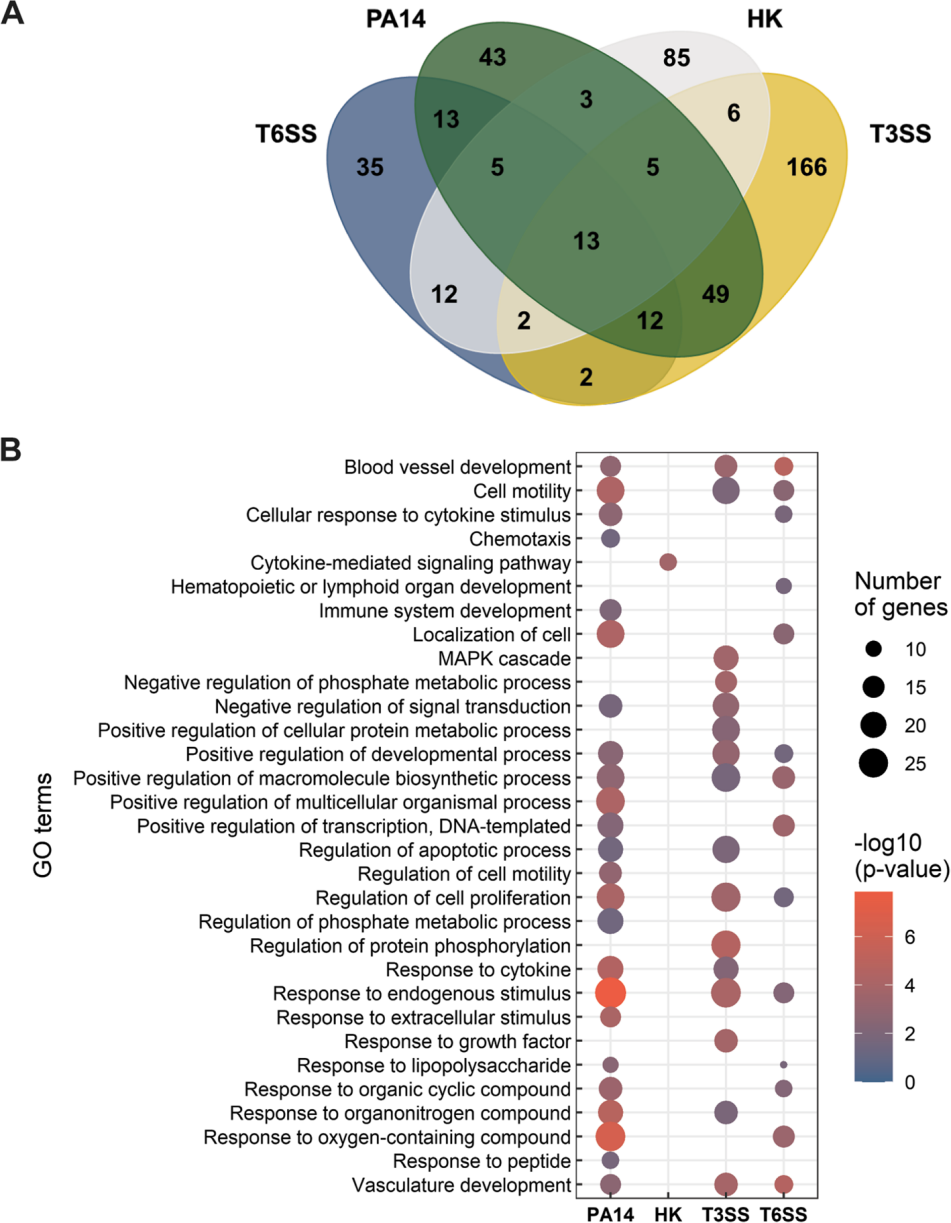
Comparison of transcriptomic and functional response in response to T3SS and T6SS *P. aeruginosa* mutants. **A** Venn diagram of differentially expressed epithelial genes. Only genes with a *p*-value ≤ 0.05, fold change > 2, and average FPKM difference > 7 were analyzed as differentially regulated genes. **B** Comparison of GO term analysis of “biological processes” of epithelial cells positively enriched in response to *P. aeruginosa* PA14 (PA14), heat-killed *P. aeruginosa* PA14 (HK), *P. aeruginosa* PA14::pscC (T3SS), and *P. aeruginosa* PA14 ΔHSI-II : : III (T6SS). Only GO terms with *p* ≤ 0.05 and number of genes associated with GO term > 5 is shown. The node color indicates the significance of functionally enriched GO terms in each group. The node size correlates with number of genes associated with functionally enriched GO terms

*P. aeruginosa* manipulates host responses by directly injecting its effectors into epithelial cells using T3SS and T6SS [14, 15]. We hypothesized that the changes in the transcriptomic response to *P. aeruginosa* are in part due to the action of T3SS and T6SS effector proteins. To test this hypothesis, lung epithelial cells were infected for 1 h with *P. aeruginosa* mutants with defective T3SS and T6SS*. P. aeruginosa* PA14*::pscC* transposon mutant [33] was used to study the effect of T3SS. This mutant has a transposon insertion in the *pscC* gene, an outer-membrane secretion component of *P. aeruginosa* T3SS [34]. *P. aeruginosa* has three T6SS loci responsible for various functions of this system. H1-T6SS is thought to be associated with *P. aeruginosa* interactions with prokaryotic cells, while H2-T6SS and H3-T6SS have been reported to be involved in interactions with eukaryotic cells [15]. In addition, H2-T6SS and H3-T6SS are known to functionally compensate for each other in animal models of *P. aeruginosa* infection [35]. Therefore, the *P. aeruginosa* PA14 ΔHSI-II:: III mutant [35] was used to study the effect of T6SS on epithelial cell response. This mutant harbors deletion of the HSI-II and HSI-III gene loci of *P. aeruginosa,* which are essential for forming the T6SS needle and secretion of effector proteins [27]. As a control, heat-killed inactivated *P. aeruginosa* was used. Heat-killed *P. aeruginosa* can no longer actively use injectosomes and secrete effector proteins directly into the epithelial cells, however, heat-killed bacteria still contains surface components capable of binding to eukaryotic cells and triggering immune responses such as lipopolysaccharide or flagellin. Differential gene expression of epithelial cells in response to live, heat-killed, or *P. aeruginosa* mutants with defective T3SS or T6SS were performed (Table S1) and Cluster analysis showed gene expression patterns common or specific for each group (Fig. 3A). A total of 13 genes were differentially regulated in all groups compared to the non-infected mock control (Fig. 3A). Differentially regulated genes included the cytokines/chemokines *CXCL8*, *CXCL2*, *CXCL3*, *CCL20*, and immediate-early response genes *EGR1*, *FOS*, *KLF6*, *NR4A1*, and *IER2* (Table 3). Differential gene expression patterns of EGR1, FOS and CXCL8 genes were also confirmed using qRT-PCR (Figure S2). While the expression levels of these genes varied in each group, all were differentially expressed in response to *P. aeruginosa* independently of bacterial viability and presence of T3SS or T6SS. Interestingly, most of the genes differentially regulated in each condition were unique to that condition, suggesting that infection with each strain or mutant is characterized by a distinct transcriptional signature (Fig. 3A).

**Table 3:** Common DEG’s in response to parental strain, heat-killed, T3SS, or T6SS mutant *P. aeruginosa.* Fold-change of common DEG’s compared to non-infected mock control epithelial cells are shown

Gene symbol	PA14	HK	T3SS	T6SS
*EGR1*	28.41	9.08	16.64	31.27
*FOS*	17.47	7.23	8.93	36.46
*NR4A1*	3.38	2.36	2.42	3.16
*KLF6*	3.22	2.05	2.43	2.73
*IER2*	2.68	2.05	2.03	2.78
*U1_8*	83.98	41.99	60.44	57.08
*RNVU1-18*	14.23	3.36	8.22	13.02
*CCL20*	4.15	2.64	2.92	3.86
*CRABP2*	4.1	2.11	4.61	2.09
*CXCL3*	3.2	3.49	2.04	3.17
*PRDM1*	3.1	2.38	3.23	2.77
*CXCL2*	3.01	3.12	2.83	3.99
*CXCL8*	2.57	2.26	2.06	3.24

To better characterize these unique transcriptomic signatures, GO term analysis was performed on the genes upregulated in response to each treatment. Only genes associated with the cytokine-mediated signaling pathway were enriched in heat-killed *P. aeruginosa* infected epithelial cells compared to mock control (Fig. 3B). The biological GO terms shared in response to *P. aeruginosa* parental strain*,* T3SS, and T6SS mutants included GO terms associated with cell motility, positive regulation of developmental process, response to endogenous stimulus, and regulation of cell proliferation compared to non-infected cells (Fig. 3B). Interestingly, among the enriched GO terms, the regulation of the apoptotic process was only enriched in epithelial cells in response to *P. aeruginosa* parental strain PA14 and the T3SS mutant, but not with the heat-killed PA14 nor the T6SS mutant (Fig. 3B). This result suggests the potential effect of T6SS on the cellular apoptosis process inside the host. T3SS of *P. aeruginosa* secretes exotoxins involved in the modulation of cytoskeleton rearrangement [14]. Accordingly, GO terms associated with cell localization were only enriched in epithelial cells infected with the parental *P. aeruginosa* strain and the T6SS mutant, but not with the heat-killed PA14 or T3SS mutant (Fig. 3B).

**Fig. 4:**
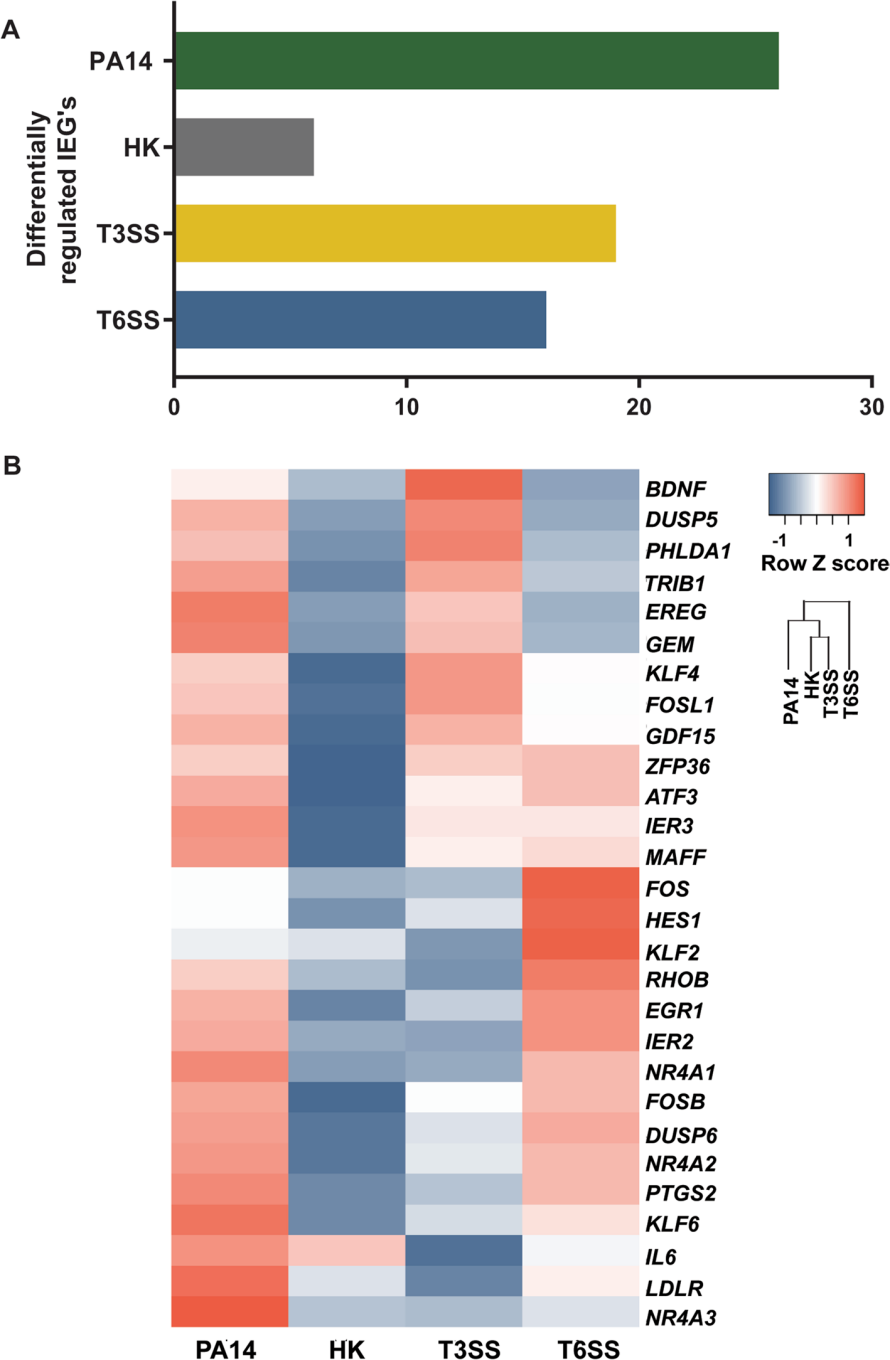
Differential expression of IEG’s during contact with T3SS and T6SS defective *P. aeruginosa* strains. **A** Total number of differentially regulated immediate-early response genes Only genes with a *p*-value ≤ 0.05, fold change > 2, and average FPKM difference > 7 were analyzed as differentially regulated genes. **B** Heatmap of differentially regulated early response genes in epithelial cells incubated with *P. aeruginosa* PA14 (PA14), heat-killed *P. aeruginosa* PA14 (HK), *P. aeruginosa* PA14::pscC (T3SS), and *P. aeruginosa* PA14 ΔHSI-II : : III (T6SS) compared to non-infected mock control

**Fig. 5:**
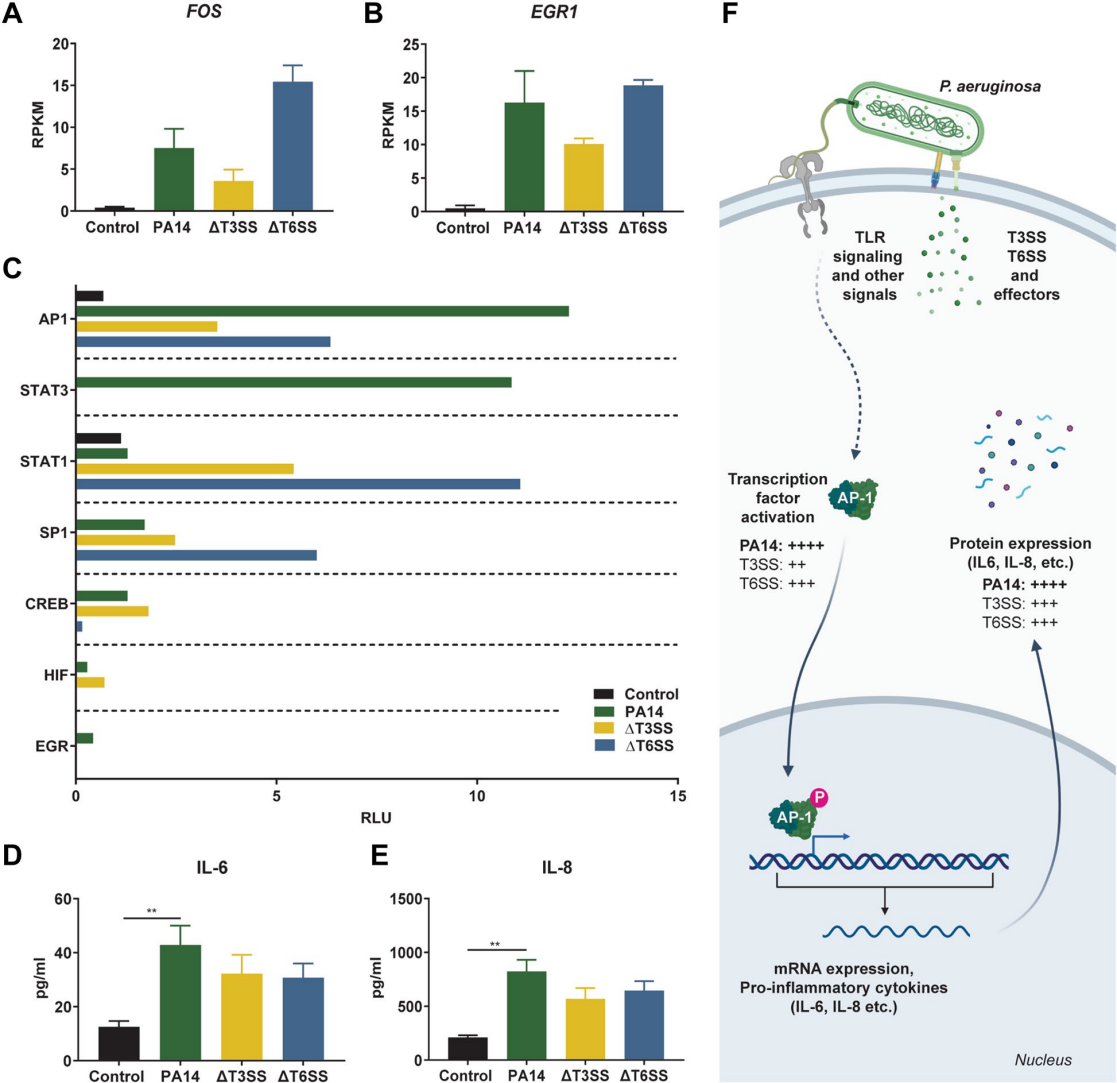
Activation of transcription factors and cytokine response in epithelial cells stimulated with T3SS, T6SS or parental strain of *P. aeruginosa*. RPKM values of **A** FOS and **B** EGR1 genes. **C** Transcription factor activation of nuclear extracts of epithelial cells incubated with *P. aeruginosa* PA14 (PA14), *P. aeruginosa* PA14::pscC (T3SS), and *P. aeruginosa* PA14 ΔHSI-II : : III (T6SS) and mock control. The values were represented as relative light units (RLU) normalized to ER (Estrogen Receptor). Cytokines **D** IL-6 and **E** IL-8 were quantified from supernatants of epithelial cells at 6 hrs post-infection. Kruskal Wallis test with Dunn’s multiple comparisons was performed for statistical analysis (**, *p* ≤ 0.01) **F** Graphical illustration of epithelial cell response to *P. aeruginosa* infection and the effect of injectosomes

The transcriptomic response of type II pneumocytes to *P. aeruginosa* was characterized by the up-regulation of IEGs at 1 h post-infection (Fig. 2A). The expression of these genes can be stimulated by a variety of internal and external signals [11]. We therefore explored whether injectosomes of *P. aeruginosa* have a specific role in the upregulation of IEGs by comparing the total number of differentially regulated IEGs in each group (Fig. 4A). Epithelial cells incubated with the PA14 parental strain *P. aeruginosa* had the highest, while heat-killed *P. aeruginosa* had the lowest number of differentially upregulated IEGs (Fig. 4A). Epithelial cells infected with *P. aeruginosa* deficient of T3SS or T6SS still led to the differential regulation of IEGs, although to a lower extend. Interestingly, the expression patterns of several IEGs were unique to T3SS and T6SS mutants compared to the PA14 parental strain (Fig. 4B). In the absence of T3SS, especially the induction of *EGR1*, and *FOS* expression was lower than the parental strain in transcriptomic analysis (Fig. 4B, Table 3). During infection with the T6SS mutant, the expression of the *FOS* and *EGR1* genes increased in epithelial cells compared to infection with the parental strain (Fig. 4B, Fig. 5A, Fig. 5B, Table 3). These results suggest that T3SS is associated with downregulation of the expression of IEGs at the center of the response elicited by epithelial cells, such as *EGR1* and *FOS* (Fig. 1C).

### T3SS and T6SS trigger activation of different transcription factors during *P. aeruginosa* infection

The expression of IEGs is controlled by transcriptional regulators, which are activated by cell-intrinsic and extrinsic signals upon infection [11]. A transcription factor activation assay was therefore performed to understand the effect of *P. aeruginosa* injectosomes on the activation of transcription factors. To do that, activation of transcription factors predicted to be upstream regulators of *EGR1* and *FOS* was measured in nuclear extracts of epithelial cells infected with PA14, the T3SS and T6SS mutants (Table 4, Fig. 5C). AP1 transcription factor was activated in the parental strain*,* and the activation level of this transcription factor was lower in T3SS and T6SS mutants (Fig. 5C). On the other hand, STAT3 was only activated during infection with the parental *P. aeruginosa* strain. Interestingly, STAT1 and SP1 activation increased in the T6SS mutant compared to the parental strain and the T3SS mutant (Fig. 5C). While the EGR1 encoding gene was the most differentially upregulated gene in epithelial cells upon infection with *P. aeruginosa,* the activation of the EGR transcription factor was not observed at the protein level, highlighting the importance of performing this type of analysis at both the mRNA and the protein level (Fig. 5C). To take these observations a step further, we also quantified the production of IL-6 and IL-8, two cytokines whose expression is controlled by EGR1 and FOS. Since no cytokines were detectable in culture supernatants of infected cells 1 h post-infection (data not shown), we extended the duration of infection to 6 h by washing the cells to remove non-adherent bacteria and replacing the medium with fresh medium containing antibiotic to prevent bacterial overgrowth and cell dealth. Six hours post-infection, we detected a significant increase in IL-6 and IL-8 secretion in cells infected with PA14 (Fig. 5D and E). We did not detect a significant increase in the secretion of either cytokines in cells infected with the T3SS and T6SS mutants, suggesting that both of these virulence systems play a role in triggering IL-6 and IL-8 production during infection. Overall, these data indicate that T3SS and T6SS affect the activation levels of transcription factors that are important for induction and control of IEG expression during infection with *P. aeruginosa* in epithelial cells, and that this activation results in changes in the secretion of proinflammatory mediators. A model with an example of the AP-1 signaling cascade and its downstream effects is shown in Fig. 5F.

**Table 4:** Predicted upstream regulators of *EGR1* and/or *FOS* genes in epithelial cells infected with *P. aeruginosa*. Selected upstream regulators were identified based on genes that were differentially regulated in epithelial cells after one hour infection with *P. aeruginosa*. Activation z-score shows predicted activation states of upstream regulators. The further the activation z-score from zero, the more likely that observed directionality of the target genes are consistent with the upstream regulator

Upstream regulator	Activation z-score	*P* value
FOS	2.33	6.33 × 10^–13^
JUN	2.555	2.73 × 10^–15^
STAT3	3.553	1.71 × 10^–14^
STAT1	2.207	6.38 × 10^–7^
SP1	2.530	3.79 × 10^–23^
CREB	2.953	1.25 × 10^–11^
HIF1A	2.521	4.63 × 10^–13^
EGR1	3.093	3.98 × 10^–11^

## Discussion

Airway epithelial cells are the first barrier against pathogens and one of the first responders during respiratory infections. Previous studies provided substantial information on epithelial cell responses during *P. aeruginosa* infection using RNA-sequencing and microarray technologies [12, 13, 36]. Here, we performed RNA-sequencing to study the response of airway epithelial cells during the early stages of infection with *P. aeruginosa* PA14*,* T3SS, and T6SS mutants to understand the role of the pathogen’s injectosomes in this process. This study identified various signaling pathways differentially controlled by T3SS and T6SS during airway epithelial cell infection in vitro and provides additional insights into the role of these two important virulence factors.

During lung infection, the initial response of airway epithelial cells to bacterial pathogens is critical to drive the recruitment of innate immune cells to the site of infection. As the infection progresses, the transcriptional landscape becomes more complex as various populations of immune cells are recruited and *P. aeruginosa* causes damage, inducing among other things, cell death. To gain insights into the initial changes in gene expression that occur upon infection with *P. aeruginosa* in A549 type II pneumocytes, we selected a short incubation timeframe (1 h). RNA-sequencing analysis identified 143 genes differentially expressed in A549 cells based on *p-*value ≤ 0.05, fold change > 2, and average FPKM difference > 7 criteria one hour post-infection with *P. aeruginosa*. Previously, high-density DNA microarray was used to identify differentially regulated genes in A549 cells [12]. As a part of that study, *P. aeruginosa* PAK strain and cells were incubated with bacteria for 3 h and only 24 genes were found to be differentially regulated. Another similar microarray study using *P. aeruginosa* PA103 to infect 9HTEo^-^ cells for 3 h reported differential regulation of 46 genes [36]. More recently, Balloy et al. performed a transcriptomic study using human airway epithelial cells from bronchial biopsies infected with *P. aeruginosa* PAK strain using RNA-sequencing at different time points of infection [13]. Some of the differentially regulated genes and pathways identified in these studies coincide with the findings of this current study, such as TNF signaling, *CCL20*, *ZFP36* (*TTP*), *IL6* or *FOS*, however there were also some discrepancies. Differences in bacterial strains or epithelia cell lines, timing of the experiment, multiplicity of infection, sensitivity of the techniques used, and stringency of differential expression analysis likely account for some of the differences observed between these studies.

Here, we observed that infection of epithelial cells with *P. aeruginosa* leads to the induction of genes associated with MAPK, IL-17, and TNF signaling pathways (Fig. 1C). The induction of the MAPK pathway and proinflammatory cytokines such as TNFα, IL-6 and IL-17 was previously observed during *P. aeruginosa* infection [36–40]. In addition, *P. aeruginosa* infection of type II pneumocytes was characterized by the upregulation of the expression of the proinflammatory cytokines/chemokines CXCL8, CXCL2, CXCL3, and CCL20 encoding genes (Table 3). The induction of these proinflammatory cytokines/chemokines can be mediated by immunogenic components of *P. aeruginosa,* such as LPS and flagella, that induce TLR4 and TLR5 signaling in epithelial cells, respectively. It was previously demonstrated that *P. aeruginosa* flagellin alone could stimulate CXCL8, CXCL2, and CCL20 production by human airway epithelial cells [41, 42]. In addition, *P. aeruginosa* LPS can induce *CXCL3* and *CCL20* gene expression in human leukocytes and cause induction of CXCL2 in mouse corneas [43, 44]. Among the differentially expressed cytokines, the expression of the IL-6 encoding gene in epithelial cells was lower in the absence of T3SS relative to the parental *P. aeruginosa* strain (Fig. 4B). This result was confirmed at the protein level as well (Fig. 5D). IL-6 was identified as one of the hub proteins and as upstream regulator of differentially expressed genes during *P. aeruginosa* infection (Fig. 1C, 2B). During in vivo* P. aeruginosa* infection, the *Il6* gene was shown to be one of the highest upregulated genes in mice lungs [45]. While multiple bacterial components of *P. aeruginosa* contribute to the induction of IL-6 responses in epithelial cells [42, 46], it was previously reported that the T3SS and its effector toxins alone can induce IL-6 secretion in human epithelial cells [47]. Overall, the data obtained in this study supports prior observations in the field on the induction of proinflammatory cytokines, and in particular, IL-6 in response to *P. aeruginosa*.

In this study, upon one-hour post-infection with *P. aeruginosa*, 17.5% of upregulated genes were IEGs. Some of these IEGs were also predicted to have a role as upstream regulators of the differentially expressed genes (Fig. 2B). Mitogens, growth factors or cell stressors are often involved in triggering initial induction of IEGs [11, 16, 48]. While bacterial components such as LPS are known to stimulate IEGs expression [49], the role of *P. aeruginosa* T3SS and T6SS in this process is still unknown. Infection with T3SS and T6SS mutants led to differences in the induction of IEGs, suggesting that each system induces a unique transcriptomic signature. These signatures were associated with differences in the activation of transcription factors such as AP1, STAT1, and SP1.

IEGs are associated with the activation of proinflammatory response genes and pathways [50]. Among IEGs, *EGR1* was the most differentially regulated IEG upon infection with *P. aeruginosa* (Fig. 2A, Table 1)*.* Elevation in the expression of the *EGR1* gene was previously reported in pulmonary diseases [51, 52] and bacterial infections, including *P. aeruginosa* [53–55]. An increase in *EGR1* expression is linked to the induction of various inflammatory mediators such as IL-6, IL-8, IL-1β, and TNF-α [53, 56–60]. Recently, the role of *EGR1* in host defense against *P. aeruginosa* was shown using the Egr-1-deficient mouse model [54]. Egr-1-deficiency was associated with bacterial clearance and reduced mortality, which might be linked to reduced systemic inflammation [54]. It was also previously shown that Egr-1 induction in epithelial cells depends on bacterial viability and contact with the cells [53]. In line with this study, we showed that *EGR1* gene expression was decreased in response to heat-killed *P. aeruginosa* compared to live bacteria (Fig. 4, Table 3)*.* Furthermore, the expression of the *EGR1* gene was lower in the T3SS mutant strain of *P. aeruginosa* compared to the parental strain (Fig. 4, Table 3)*.* These results suggest that *EGR1* gene expression during the early phase of infection with *P. aeruginosa* depends on bacterial viability and the T3SS. Similar observations have been made in other Gram-negative bacteria in which T3SS was shown to play a role in the induction of *EGR1* expression in epithelial cells [61–63].

The second most differentially upregulated IEG upon *P. aeruginosa* infection was the *FOS* gene (Fig. 2A, Table 1)*.* C-Fos is part of the AP-1 transcription factor complex associated with inflammatory pathways crucial for initial host-response against pathogens [64, 65]. The *FOS* gene was shown to be upregulated upon *P. aeruginosa* infection [36, 55]. In line with these studies, upon infection with *P. aeruginosa,* the *FOS* gene was upregulated compared to the non-infected mock-control (Table 1, Fig. 1). Bacterial viability and the absence of the T3SS in *P. aeruginosa* negatively affected the expression of the *FOS* gene (Table 3, Fig. 5A). In addition, the activation of the AP-1 transcription factor was lower in epithelial cells infected with the *P. aeruginosa* T3SS mutant (Fig. 5C). These results are in line with evidence that the T3SS effector ExoU is essential in the upregulation of *FOS* expression in epithelial cells [36]. Both *EGR1* and *FOS* gene expression increased in the T6SS mutant compared to parental *P. aeruginosa* infected epithelial cells. These results might be in part due to the effect of the regulation of the upstream activators of *EGR1* and *FOS* or the effect of differential regulation of other bacterial components in mutant strains. In the future, mechanistic studies with reporter and KO strains can likely elucidate the role of the T3SS and T6SS on EGR1 and FOS regulation. Additional insights could also be obtained by measuring the bacterial transcriptome response during infection. To maintain high epithelial cell viability during infection, this study was performed with a relatively low multiplicity of infection and the cells were washed prior to being saved in RNA-protect. Therefore, we did not recover enough bacterial RNA and generate sufficient reads to be able to characterize the transcriptomic response of *P. aeruginosa* and its injectosome mutants during infection. Modification of the experimental setup to separate bacterial from eukaryotic cells or mRNA would likely enable this type of dual RNA sequencing approach for this in vitro setting [45, 66].

Overall, this study sheds light on the early host responses differentially regulated in epithelial cells during infection with *P. aeruginosa* and the distinct role of T3SS and T6SS in this response. Transcriptomics, together with transcription factor activation assays and cytokine measurements highlighted the role of T3SS and T6SS in up-regulating the secretion of proinflammatory mediators such as IL-6 and IL-8 in response to *P. aeruginosa* infection (Fig. 5F). Future studies need to examine the expression of genes as well as proteins and their activation state overtime to better understand the epithelial cell response during *P. aeruginosa* infection both in vitro and in vivo. In addition, future work is required to determine the potential of therapeutic interventions targeting early response genes, such as EGR1 and FOS, or T3SS of *P. aeruginosa* to alleviate the disease burden of patients infected with *P. aeruginosa*.

## Conclusions

By comparing the transcriptional profiles of epithelial cells during infection with wild-type, and T3SS, T6SS deficient *P. aeruginosa* mutants, we show that immediate-early response genes are differentially regulated in epithelial cells during the first hour of infection with *P. aeruginosa.* This study illustrates that T3SS and T6SS deficient mutants lead to two distinct expression profiles in epithelial cells compared to the wild-type strain, and identified key regulators regulated by T3SS and T6SS.

## Methods

### Bacterial strains

*P. aeruginosa* PA14 [25], *PA14::pscC* (PA14 transposon mutant ID: 29,579) [33] and PA14 ΔHSI-II:: III strains were used in this study [35]. All *P. aeruginosa* strains were grown in 3 ml of Miller’s lysogeny broth (LB) overnight at 37 °C. Overnight cultures were diluted 1:100 in fresh LB and grown until the cultures reached OD_600_ = 0.3.

### Epithelial cell culture

Human alveolar basal epithelial adenocarcinoma A549 cells were obtained from the American Type Culture Collection (ATCC). The cells were cultured using F-12 K medium supplemented with 10% v/v fetal bovine serum (FBS) (Corning, 10–025-CV), 50 IU/mL penicillin, and 50 μg/mL streptomycin (Corning, 30001Cl) at 37 °C in 5% CO_2_.

### Infection of epithelial cells

Epithelial cells were seeded in T-75 cell culture flasks (9 × 10^6^ cells/flask) (Greiner Bio-one. 658,175) or 6 well plates (1 × 10^6^ cells/flask) (Greiner Bio-one, 657,165) a day before the experiment. *P. aeruginosa* PA14, *PA14::pscC or* PA14 ΔHSI-II:: III strains were grown as previously described. The bacterial culture was diluted to a multiplicity of infection of 10 using F-12 K medium supplemented with 10% v/v FBS. The infection dose was validated by serially diluting and plating the dose on Lysogeny Agar (LA) plates. For heat-killed control, *P. aeruginosa* PA14 grown as described above and was heat-inactivated for 1 h at 60 °C prior to get in contact with epithelial cells. Epithelial cells were washed three times, with 10 ml of phosphate buffered saline (PBS) (Corning, 21–040-CV). The bacterial suspension was added to epithelial cells and centrifuged at 167.8 × *g* for 5 min to promote bacterial attachment. Flasks were then incubated for 1 h at 37 °C in 5% CO_2_. For the non-infected mock control, epithelial cells were treated as described above and incubated with F-12K with 10% v/v FBS. Experiments were performed in independent triplicates for each condition, and the same batch of serum was used throughout the study to avoid batch-to-batch variability. Epithelial cell viability assays were performed using alamarBlue (Thermo Fisher, DAL1025) at 1 h post-infection. The epithelial cells were washed with1 ml of 1 × PBS three times. A549 cells were lysed using 0.5% v/v Triton X-100 (Sigma-Aldrich, T8787) in 1X PBS as a negative control. The cells were incubated with F-12 K medium with 10% v/v FBS (Corning, 10–025-CV) and 10% v/v alamarBlue (Thermo Fisher, DAL1025) for 5 h. The fluorescence signal was detected by using SpectraMax i3 (Molecular Devices LLC).

### RNA preparation

Epithelial cells were incubated with *P. aeruginosa* parental strain, heat-killed, T3SS mutant, T6SS mutant or medium alone for one-hour. Then these cells were washed with 10 ml of PBS (Corning, 21–040-CV) three times. Cells were scraped off using a cell scraper in 1 ml of PBS and centrifuged at 16,200 × *g* for 1 min. The pellets were resuspended in 0.5 ml of RNAprotect cell reagent (Qiagen, 76,526) and stored at -80 °C. Qiagen RNeasy Mini Kit (Qiagen, 74,104) was used for RNA extraction. The samples were thawed and centrifuged at 9,600 × *g* for 3 min. The pelleted cells were resuspended in 400 µl of 1 mg/ml of TE lysozyme (1 mg lysozyme in 1 ml of 10 mM/1 mM Tris–EDTA buffer) and incubated for 10 min at room temperature. The RNA was extracted using the RNeasy mini spin column based on the manufacturer’s instructions. Any remaining DNA was degraded using the RNase-free DNase set (Qiagen, 79,254) according to the manufacturer’s instructions. The DNase was removed from the samples using the RNeasy mini spin column (Qiagen, 74,104) and purified RNA was eluted in 50 µl of RNAse-free water (Qiagen, 74,104). The samples were confirmed to be DNA-free by quantitative PCR (qPCR) as described below. The amount of RNA in samples was measured using a Spectramax i3x (Molecular Devices), and the integrity of the samples was characterized using an Agilent 2100 Bioanalyzer. The RNA integrity number of the samples used for RNA-sequencing was greater than or equal to 8.

### Library construction, RNA sequencing, and analysis

RNA samples were depleted of rRNA using the human RiboMinus kit (Thermo Fisher Scientific). The samples were converted into Illumina sequencing libraries with the ScriptSeq v2 RNA-Seq Library Preparation Kit (Epicentre, Illumina). The libraries passed standard quality control PCR and were sequenced using an Illumina HiSeq1500 at Marshall University (2 × 75 bp reads). Three biological replicates were sequenced in each group.

Resultant reads were trimmed and aligned onto the human UCSC hg38 reference genome (GCA_000001405.27) using CLC Genomics Workbench, version 9.5.4 (Qiagen). After trimming, there were on average 19,411,441 ± 1,447,196 reads on each sample. Reads were mapped against the reference genome with the following settings: mismatch cost = 2, insertion cost = 3, deletion cost = 3, length fraction = 0.8, similarity fraction = 0.8. The depth of coverage were 21,312 gene reads on average per sample. Fold changes in gene expression, and statistical analyses were performed using the extraction of differential gene expression (EDGE) test as implemented in CLC Genomics, which is based on the Exact Test [67, 68]. Differential gene expressions of infected A549 cells were determined by comparison to non-infected mock A549 cell controls (Table S1). *P*-values calculated by EDGE test without correction. Genes with differences in gene expression with a *p-*value ≤ 0.05, fold change > 2, and average FPKM difference > 7 were used for analysis. Ribosomal proteins and RNA were discarded from analysis. The immediate-early response gene-set used in this manuscript was generated using previous literature (Table 2).

### Functional analysis

Functional enrichment of differentially expressed genes was performed using WebGestalt to determine nonredundant biological processes [69]. Statistically enriched Gene Ontology (GO) terms under “biological process” with Bonferroni correction *p*-value ≤ 0.05 were identified. The redundancy of functional GO terms was reduced using the affinity propagation method implemented in WebGestalt. The upstream regulator analysis of differentially regulated genes was performed by Ingenuity Pathway Analysis (IPA, QIAGEN Inc.) [32]. R statistical software v3.5.2 was used to visualize data using volcano plots and enriched GO Term comparisons for multiple groups [70]. Heatmaps were generated using Heatmapper [71].

### Network analysis

The protein–protein interaction network information of differentially expressed genes during PA14 infection was retrieved from STRING V11.0 [18]. The generated protein interaction network was reconstructed using Cytoscape software version 3.8 [72]. The protein nodes with a high degree of connectivity were calculated by Cytoscape network analysis. KEGG pathway enrichment analysis was made using STRING V11.0 [18].

### qRT-PCR analysis

Epithelial cells were seeded in 12 well plates (4 × 10^5^ cells/flask) (Costar, 3513) a day before the experiment. *P. aeruginosa* PA14, *PA14::pscC or* PA14 ΔHSI-II:: III strains were grown as previously described. The bacterial culture was diluted to a multiplicity of infection of 20 using F-12 K medium supplemented with 10% v/v FBS. The cells were infected and RNA was isolated after 1 h of infection from each sample as described above. Absence of DNA was confirmed by performing qPCR on 20 ng of RNA using the following primer set: RPS13 F (CGAAAGCATCTTGAGAGGAACA) and RPS13 R (TCGAGCCAAACGGTGAATC) [73]. cDNA synthesis was performed using Moloney murine leukemia virus (MMLV) reverse transcriptase (Promega, PR-M1705) according to manufacturer’s instructions using 250 ng of RNA and gene-specific reverse primers for each target. qPCR amplification was performed using SYBR Green PCR master mix (Applied Biosystems, 4,309,155) according to the manufacturer's instructions. Three technical replicates were run per gene target per sample on a StepOnePlus qPCR thermocycler (Applied Biosystems). Gene expression was normalized to that of the *RPS13* reference gene using the 2^−ΔΔCT^ method [74]. The following primer sequences were used in this study: EGR1 F (CCTGACATCTCTCTGAACAACG) EGR1 R (GGGAAAAGCGGCCAGTATAG) [55], FOS F (CCAACCTGCTGAAGGAGAAG), FOS R (AGATCAAGGGAAGCCACAGA) [75], IL6 F (CCCACCGGGAACGAAAGAG). IL6 R (CAGGGAGAAGGCAACTGGAC), CXCL8 F (CACTGCGCCAACACAGAAAT) CXCL8 R (AAGTTTCACTGGCATCTTCACT), RPS13 F (CGAAAGCATCTTGAGAGGAACA) and RPS13 R (TCGAGCCAAACGGTGAATC) [73].

### Transcription factor activating profiling array

The epithelial cells were infected with *P. aeruginosa* mutants, as described above. At 1 h post-infection, the nuclear extracts were obtained using a nuclear extraction kit (Signosis, SK-0001) following the manufacturer’s instructions. Triplicates for each condition were obtained by completing three independent experiments. The amount of protein in each extract was measured using both the NanoDrop Protein quantification instrument (Thermo Fisher Scientific) and using a bicinchoninic acid assay (BCA) total protein kit (Thermo Fisher Scientific, 23,225). Triplicates for each condition were combined and the activation level of 48 different transcription factors in each condition was measured using the Transcription Factor Activation Profiling Plate Array I (Signosis, FA1001) according to the manufacturer’s instructions. Briefly, 10 µg nuclear extracts were incubated with biotin-labeled probes encoding TF DNA-binding site consensus sequences from 30 min at room temperature. The unbound probes were washed away using the provided isolation column. The remaining probes were eluted and hybridized to complementary sequences in provided 96-well hybridization plate at 42 °C overnight. The plates were washed, blocked, and the luminescence signal measured on Synergy™ HTX Multi-Mode Microplate Reader (BioTek). The values were normalized to (ER Estrogen Receptor).

### Cytokine analysis

For cytokine analysis, epithelial cells were grown in 12 well plates (4 × 10^5^ cells/flask) as previously described. The bacterial culture was diluted to a multiplicity of infection of 20:1 using F-12 K medium supplemented with 10% v/v FBS. The cells were infected for 1 h as described above. After 1 h incubation, cells were washed 3 times with 1X PBS and the medium was replaced by F12K with 10% v/v FBS and gentamicin 300 μg/mL. Cells were incubated an additional 6 h, then supernatant was collected and preserved for cytokine analysis at -80 °C. IL-6 and IL-8 quantification was performed using the Human Luminex Discovery Assay (R&D systems, LXSAHM-20) according to manufacturer’s instructions. The samples were run on a Magpix (Luminex) instrument to detect cytokine levels. Cytokine concentration values below the limit of detection were arbitrary setup to 0 for statistical analysis and graphical representation.

### Statistical analysis

Statistical analysis of transcriptomic data was performed using differential gene expression (EDGE) test as implemented in CLC Genomics, which is based on the Exact Test [67, 68]. The statistical packages integrated in GO term [69], IPA [32], and STRING V11.0 [18] analyses were used for in silico functional and network data analyses. Data comparisons for more than two groups were performed using ordinary one-way analysis of variance (ANOVA) followed by Tukey’s multiple comparisons test. Dunnet’s multiple comparisons test was used when multiple groups were compared to control for normally distributed data. Kruskal–Wallis test with Dunn’s multiple comparison test was used for nonparametric data. All statistical analysis were done by using GraphPad Prism 9.2.0 (San Diego, California USA).

## Supplementary Information

**Additional file 1:** **Figure S1. **Cell viability of epithelial cells one-hour post-infection with *P. aeruginosa. *Cell viability of uninfected, dead or *P.aeruginosa* PA14 infected A549 epithelial cells. Relative fluorescence intensity was calculated by subtracting the background fluorescence signal. Experiments were performed in independent triplicates for each condition. Samples were compared to negative control group using One-way ANOVA followed by Dunnet’s multiple comparisons test for statistical analysis. Error bars indicate standard deviation. The asterisks show statistical significance: *p* ≤ 0.0001. **Figure S2.** qRT-PCR of selected genes in response to live and heat-killed PA14, and live T3SS and T6SS *P.aeruginosa* mutants. qRT-PCR analysis of **A** EGR1, **B** FOS, **C** IL6, and **D** CXCL8 relative fold change compared to RPS13 housekeeping gene. Analysis was performed using three biological replicates with three technical replicates. Ordinary One-way ANOVA with Tukey’s multiple comparison tests was performed for statistical analysis (*, *p* ≤ 0.05, **, *p* ≤ 0.01). **Table S1.** Analyzed RNAseq data. Each tab in the file lists the number of reads, RPKM, fold changes, *p-*values, annotations, and other relevant information for each comparison performed in this study.

**Additional file 2.**  

## Data Availability

The raw RNA sequencing read datasets generated in this study are available in NCBI Read Archive (SRA) repository with SRA Bioproject number PRJNA791600 (https://dataview.ncbi.nlm.nih.gov/object/PRJNA791600). Analyzed data are provided in the supplementary material.

## Abbreviations

*P.** aeruginosa*: *Pseudomonas aeruginosa*
T3SS: Type Three Secretion System
T6SS: Type Six Secretion System
IEG: Immediate-Early Response Gene
LPS: Lipopolysaccharides
CF: Cystic Fibrosis

## References

[1] Gellatly SL, Hancock REW (2013). *Pseudomonas aeruginosa*: New insights into pathogenesis and host defenses. Pathog Dis.

[2] Sadikot RT, Blackwell TS, Christman JW, Prince AS. Pathogen-host interactions in *Pseudomonas aeruginosa* pneumonia. Am J Respir Crit Care Med. 2005;171:1209–23. 10.1164/rccm.200408-1044SO PMC2718459 15695491

[3] Cystic Fibrosis Foundation Patient Registry. 2019 Annual Data Report. 2019.

[4] Frederiksen B, Koch C, Høiby N (1997). Antibiotic treatment of initial colonization with *Pseudomonas aeruginosa* postpones chronic infection and prevents deterioration of pulmonary function in cystic fibrosis. Pediatr Pulmonol.

[5] U.S Department of Health and Human Services Center for Disease Control and Prevention. Antibiotic Resistance Threats in the United States, 2019. 2019.

[6] Moradali MF, Ghods S, Rehm BHAA (2017). *Pseudomonas aeruginosa* Lifestyle: A Paradigm for Adaptation, Survival, and Persistence. Front Cell Infect Microbiol.

[7] Whitsett JA (2002). Intrinsic and innate defenses in the lung: Intersection of pathways regulating lung morphogenesis, host defense, and repair. J Clin Invest.

[8] Lewis BW, Patial S, Saini Y (2019). Immunopathology of Airway Surface Liquid Dehydration Disease. J Immunol Res.

[9] Hariri BM, Cohen NA. New insights into upper airway innate immunity. Am J Rhinol Allergy. 2016;30(5):319-23. 10.2500/ajra.2016.30.4360 PMC5013235 27657896

[10] Smale ST (2010). Selective Transcription in Response to an Inflammatory Stimulus. Cell.

[11] Bahrami S, Drabløs F (2016). Gene regulation in the immediate-early response process. Adv Biol Regul.

[12] Ichikawa JK, Norris A, Bangera MG, Geiss GK, Van ‘tWout AB, Bumgarner RE (2000). Interaction of *Pseudomonas aeruginosa* with epithelial cells: Identification of differentially regulated genes by expression microarray analysis of human cDNAs. Proc Natl Acad Sci U S A.

[13] Balloy V, Varet H, Dillies MA, Proux C, Jagla B, Coppée JY (2015). Normal and cystic fibrosis human bronchial epithelial cells infected with *Pseudomonas aeruginosa* exhibit distinct gene activation patterns. PLoS ONE.

[14] Hauser AR (2009). The type III secretion system of *Pseudomonas aeruginosa*: Infection by injection. Nat Rev Microbiol.

[15] Sana TG, Berni B, Bleves S (2016). The T6SSs of *Pseudomonas aeruginosa* strain PAO1 and their effectors: Beyond bacterial-cell targeting. Front Cell Infect Microbiol.

[16] Tullai JW, Schaffer ME, Mullenbrock S, Sholder G, Kasif S, Cooper GM (2007). Immediate-early and delayed primary response genes are distinct in function and genomic architecture. J Biol Chem.

[17] Uhlitz F, Sieber A, Wyler E, Fritsche-Guenther R, Meisig J, Landthaler M (2017). An immediate–late gene expression module decodes ERK signal duration. Mol Syst Biol.

[18] Szklarczyk D, Gable AL, Lyon D, Junge A, Wyder S, Huerta-Cepas J (2019). STRING v11: Protein-protein association networks with increased coverage, supporting functional discovery in genome-wide experimental datasets. Nucleic Acids Res.

[19] Svaren J, Ehrig T, Abdulkadir SA, Ehrengruber MU, Watson MA, Milbrandt J (2000). EGR1 target genes in prostate carcinoma cells identified by microarray analysis. J Biol Chem.

[20] Fu M, Zhu X, Zhang J, Liang J, Lin Y, Zhao L (2003). Egr-1 target genes in human endothelial cells identified by microarray analysis. Gene.

[21] Shaulian E, Karin M (2002). AP-1 as a regulator of cell life and death. Nat Cell Biol.

[22] Wu YE, Pan L, Zuo Y, Li X, Hong W (2017). Detecting Activated Cell Populations Using Single-Cell RNA-Seq. Neuron.

[23] Cullingford TE, Butler MJ, Marshall AK, Tham EL, Sugden PH, Clerk A (2008). Differential regulation of Krüppel-like factor family transcription factor expression in neonatal rat cardiac myocytes: Effects of endothelin-1, oxidative stress and cytokines. Biochim Biophys Acta - Mol Cell Res.

[24] Aitken S, Magi S, Alhendi AMN, Itoh M, Kawaji H, Lassmann T (2015). Transcriptional Dynamics Reveal Critical Roles for Non-coding RNAs in the Immediate-Early Response. PLoS Comput Biol.

[25] Crofford LJ (1997). COX-1 and COX-2 tissue expression: Implications and predictions. J Rheumatol.

[26] Wadleigh DJ, Reddy ST, Kopp E, Ghosh S, Herschman HR (2000). Transcriptional activation of the cyclooxygenase-2 gene in endotoxin- treated RAW 264.7 macrophages. J Biol Chem.

[27] Lee SM, Vasishtha M, Prywes R (2010). Activation and repression of cellular immediate early genes by serum response factor cofactors. J Biol Chem.

[28] Neef R, Kuske MA, Pröls E, Johnson JP (2002). Identification of the human PHLDA1/TDAG51 gene: Down-regulation in metastatic melanoma contributes to apoptosis resistance and growth deregulation. Cancer Res.

[29] Fambrough D, McClure K, Kazlauskas A, Lander ES (1999). Diverse signaling pathways activated by growth factor receptors induce broadly overlapping, rather than independent, sets of genes. Cell.

[30] Rickhag M, Teilum M, Wieloch T (2007). Rapid and long-term induction of effector immediate early genes (BDNF, Neuritin and Arc) in peri-infarct cortex and dentate gyrus after ischemic injury in rat brain. Brain Res.

[31] Jähner D, Hunter T (1991). The ras-related gene rhoB is an immediate-early gene inducible by v-Fps, epidermal growth factor, and platelet-derived growth factor in rat fibroblasts. Mol Cell Biol.

[32] Krämer A, Green J, Pollard J, Tugendreich S (2014). Causal analysis approaches in ingenuity pathway analysis. Bioinformatics.

[33] Liberati NT, Urbach JM, Miyata S, Lee DG, Drenkard E, Wu G (2006). An ordered, nonredundant library of *Pseudomonas aeruginosa* strain PA14 transposon insertion mutants. Proc Natl Acad Sci.

[34] Galle M, Jin S, Bogaert P, Haegman M, Vandenabeele P, Beyaert R (2012). The *Pseudomonas aeruginosa* type III secretion system has an exotoxin S/T/Y independent pathogenic role during acute lung infection. PLoS One..

[35] Lesic B, Starkey M, He J, Hazan R, Rahme LG (2009). Quorum sensing differentially regulates *Pseudomonas aeruginosa* type VI secretion locus I and homologous loci II and III, which are required for pathogenesis. Microbiol.

[36] McMorran B, Town L, Costelloe E, Palmer J, Engel J, Hume D (2003). Effector ExoU from the type III secretion system is an important modulator of gene expression in lung epithelial cells in response to *Pseudomonas aeruginosa* infection. Infect Immun.

[37] Dosunmu EF, Emeh RO, Dixit S, Bakeer MK, Coats MT, Owen DR (2017). The anti-microbial peptide TP359 attenuates inflammation in human lung cells infected with *Pseudomonas aeruginosa* via TLR5 and MAPK pathways. PLoS ONE.

[38] Zhang Z, Reenstra W, Weiner DJ, Louboutin JP, Wilson JM (2007). The p38 mitogen-activated protein kinase signaling pathway is coupled to Toll-like receptor 5 to mediate gene regulation in response to *Pseudomonas aeruginosa* infection in human airway epithelial cells. Infect Immun.

[39] Kube D, Sontich U, Fletcher D, Davis PB. Proinflammatory cytokine responses to *P. aeruginosa* infection in human airway epithelial cell lines. Am J Physiol - Lung Cell Mol Physiol. 2001;280:L493-502. 10.1152/ajplung.2001.280.3.L493 11159033

[40] Liu J, Feng Y, Yang K, Li Q, Ye L, Han L, et al. Early production of IL-17 protects against acute pulmonary *Pseudomonas aeruginosa* infection in mice. FEMS Immunol Med Microbiol. 2011;61(2):179–88. 10.1111/j.1574-695X.2010.00764.x 21204996

[41] Shanks KK, Guang W, Kim KC, Lillehoj EP (2010). Interleukin-8 production by human airway epithelial cells in response to *Pseudomonas aeruginosa* clinical isolates expressing type a or type b flagellins. Clin Vaccine Immunol.

[42] Parker D, Prince A (2013). Epithelial Uptake of Flagella Initiates Proinflammatory Signaling. PLoS ONE.

[43] Luan L, Patil NK, Guo Y, Hernandez A, Bohannon JK, Fensterheim BA (2017). Comparative Transcriptome Profiles of Human Blood in Response to the Toll-like Receptor 4 Ligands Lipopolysaccharide and Monophosphoryl Lipid A. Sci Rep.

[44] Khatri S, Lass JH, Heinzel FP, Petroll WM, Gomez J, Diaconu E (2002). Regulation of endotoxin-induced keratitis by PECAM-1, MIP-2, and toll-like receptor 4. Investig Ophthalmol Vis Sci.

[45] Damron FH, Oglesby-Sherrouse AG, Wilks A, Barbier M (2016). Dual-seq transcriptomics reveals the battle for iron during *Pseudomonas aeruginosa* acute murine pneumonia. Sci Rep.

[46] Raoust E, Balloy V, Garcia-Verdugo I, Touqui L, Ramphal R, Chignard M (2009). *Pseudomonas aeruginosa* LPS or flagellin are sufficient to activate TLR-dependent signaling in murine alveolar macrophages and airway epithelial cells. PLoS ONE.

[47] Park JW, Kim YJ, Shin IS, Kwon OK, Hong JM, Shin NR (2016). Type III Secretion System of *Pseudomonas aeruginosa* Affects Matrix Metalloproteinase 12 (MMP-12) and MMP-13 Expression via Nuclear Factor κb Signaling in Human Carcinoma Epithelial Cells and a Pneumonia Mouse Model. J Infect Dis.

[48] Cohen DM (1997). Mitogen-activated protein kinase cascades and the signaling of hyperosmotic stress to immediate early genes. Comp Biochem Physiol A Physiol.

[49] Raza S, Barnett MW, Barnett-Itzhaki Z, Amit I, Hume DA, Freeman TC (2014). Analysis of the transcriptional networks underpinning the activation of murine macrophages by inflammatory mediators. J Leukoc Biol.

[50] Ahmed AU, Williams BRG, Hannigan GE (2015). Transcriptional activation of inflammatory genes: Mechanistic insight into selectivity and diversity. Biomolecules.

[51] Ning W, Li CJ, Kaminski N, Feghali-Bostwick CA, Alber SM, Di YP (2004). Comprehensive gene expression profiles reveal pathways related to the pathogenesis of chronic obstructive pulmonary disease. Proc Natl Acad Sci U S A.

[52] Zhang W, Du Yan S, Zhu A, Yu Shan Zou YS, Williams M, Godman GC (2000). Expression of Egr-1 in late stage emphysema. Am J Pathol..

[53] de Klerk N, Saroj SD, Wassing GM, Maudsdotter L, Jonsson AB (2017). The host cell transcription factor EGR1 is induced by bacteria through the EGFR-ERK1/2 pathway. Front Cell Infect Microbiol.

[54] Pang Z, Raudonis R, McCormick C, Cheng Z (2019). Early growth response 1 deficiency protects the host against *Pseudomonas aeruginosa* lung infection. Infect Immun.

[55] Wu Y, Li D, Wang Y, Liu X, Zhang Y, Qu W, et al. Beta-defensin 2 and 3 promote bacterial clearance of *Pseudomonas aeruginosa* by inhibiting macrophage autophagy through downregulation of early growth response gene-1 and c-FOS. Front Immunol. 2018;9:211. 10.3389/fimmu.2018.00211 PMC5816924 29487594

[56] Reynolds PR, Cosio MG, Hoidal JR (2006). Cigarette smoke-induced Egr-1 upregulates proinflammatoiy cytokines in pulmonary epithelial cells. Am J Respir Cell Mol Biol.

[57] Chu L, Wang T, Hu Y, Gu Y, Su Z, Jiang H (2013). Activation of Egr-1 in Human Lung Epithelial Cells Exposed to Silica through MAPKs Signaling Pathways. PLoS ONE.

[58] Shi L, Kishore R, McMullen MR, Nagy LE (2002). Lipopolysaccharide stimulation of ERK1/2 increases TNF-α production via Egr-1. Am J Physiol - Cell Physiol.

[59] Hoffmann E, Ashouri J, Wolter S, Doerrie A, Dittrich-Breiholz O, Schneider H (2008). Transcriptional regulation of EGR-1 by the interleukin-1-JNK-MKK7-c-Jun pathway. J Biol Chem.

[60] Ma J, Ren Z, Ma Y, Xu L, Zhao Y, Zheng C (2009). Targeted knockdown of EGR-1 inhibits IL-8 production and IL-8-mediated invasion of prostate cancer cells throughsuppressing EGR-1/NF-κB synergy. J Biol Chem.

[61] De Grado M, Rosenberger CM, Gauthier A, Vallance BA, Finlay BB (2001). Enteropathogenic *Escherichia coli* infection induces expression of the early growth response factor by activating mitogen-activated protein kinase cascades in epithelial cells. Infect Immun.

[62] Hannemann S, Gao B, Galán JE (2013). Salmonella Modulation of Host Cell Gene Expression Promotes Its Intracellular Growth. PLoS Pathog.

[63] Kwuan L, Adams W, Auerbuch V (2013). Impact of host membrane pore formation by the *Yersinia pseudotuberculosis* type III secretion system on the macrophage innate immune response. Infect Immun.

[64] Wang A, Al-Kuhlani M, Johnston SC, Ojcius DM, Chou J, Dean D (2013). Transcription factor complex AP-1 mediates inflammation initiated by *Chlamydia pneumoniae* infection. Cell Microbiol.

[65] Seo JH, Lim JW, Kim H, Kim KH (2004). *Helicobacter pylori* in a Korean isolate activates mitogen-activated protein kinases, AP-1, and NF-κB and induces chemokine expression in gastric epithelial AGS cells. Lab Investig.

[66] Wong TY, Hall JM, Nowak ES, Boehm DT, Gonyar LA, Hewlett EL, et al. Analysis of the In *In Vivo* Transcriptome of *Bordetella pertussis* during Infection of Mice. mSphere. 2019;4:e00154-19. 10.1128/mSphereDirect.00154-19 PMC6470212 30996109

[67] Leek JT, Monsen E, Dabney AR, Storey JD (2006). EDGE: Extraction and analysis of differential gene expression. Bioinformatics.

[68] Robinson MD, Smyth GK (2008). Small-sample estimation of negative binomial dispersion, with applications to SAGE data. Biostatistics.

[69] Liao Y, Wang J, Jaehnig EJ, Shi Z, Zhang B (2019). WebGestalt 2019: gene set analysis toolkit with revamped UIs and APIs. Nucleic Acids Res.

[70] Bonnot T, Gillard M, Nagel D. A Simple Protocol for Informative Visualization of Enriched Gene Ontology Terms. Bio-101. 2019;e3429. https://www.semanticscholar.org/paper/A-Simple-Protocol-for-Informative-Visualization-of-Bonnot-Gillard/2bd842cb400f2fc651559945e54e471ad3a5914f.

[71] Babicki S, Arndt D, Marcu A, Liang Y, Grant JR, Maciejewski A (2016). Heatmapper: web-enabled heat mapping for all. Nucleic Acids Res.

[72] Shannon P, Markiel A, Ozier O, Baliga NS, Wang JT, Ramage D (2003). Cytoscape: A software Environment for integrated models of biomolecular interaction networks. Genome Res.

[73] Jacob F, Guertler R, Naim S, Nixdorf S, Fedier A, Hacker NF (2013). Careful Selection of Reference Genes Is Required for Reliable Performance of RT-qPCR in Human Normal and Cancer Cell Lines. PLoS One.

[74] Livak KJ, Schmittgen TD. Analysis of relative gene expression data using real-time quantitative PCR and the 2(-Delta Delta C(T)) Method. Methods. 2001;25(4):402-8. 10.1006/meth.2001.1262 11846609

[75] Renaud SJ, Kubota K, Rumi MAK, Soares MJ (2014). The FOS transcription factor family differentially controls trophoblast migration and invasion. J Biol Chem.

