# Supplementary material for: P. aeruginosa type III and type VI secretion systems modulate early response gene expression in type II pneumocytes in vitro
Source: BMC Genomics. 2022 May 4;23:345. doi: 10.1186/s12864-022-08554-0 (PMC9068226; doi:10.1186/s12864-022-08554-0)
Supplement: Supplementary file 1 — Additional file 1: Figure S1. Cell viability of epithelial cells one-hour post-infection with P. aeruginosa. Cell viability of uninfected, dead or P.aeruginosa PA14 infected A549 epithelial cells. Relative fluorescence intensity was calculated by subtracting the background fluorescence signal. Experiments were performed in independent triplicates for each condition. Samples were compared to negative control group using One-way ANOVA followed by Dunnet’s multiple comparisons test for statistical analysis. Error bars indicate standard deviation. The asterisks show statistical significance: p ≤ 0.0001. Figure S2. qRT-PCR of selected genes in response to live and heat-killed PA14, and live T3SS and T6SS P.aeruginosa mutants. qRT-PCR analysis of A EGR1, B FOS, C IL6, and D CXCL8 relative fold change compared to RPS13 housekeeping gene. Analysis was performed using three biological replicates with three technical replicates. Ordinary One-way ANOVA with Tukey’s multiple comparison tests was performed for statistical analysis (*, p ≤ 0.05, **, p ≤ 0.01). Table S1. Analyzed RNAseq data. Each tab in the file lists the number of reads, RPKM, fold changes, p-values, annotations, and other relevant information for each comparison performed in this study. [file 12864_2022_8554_MOESM1_ESM.docx]

**
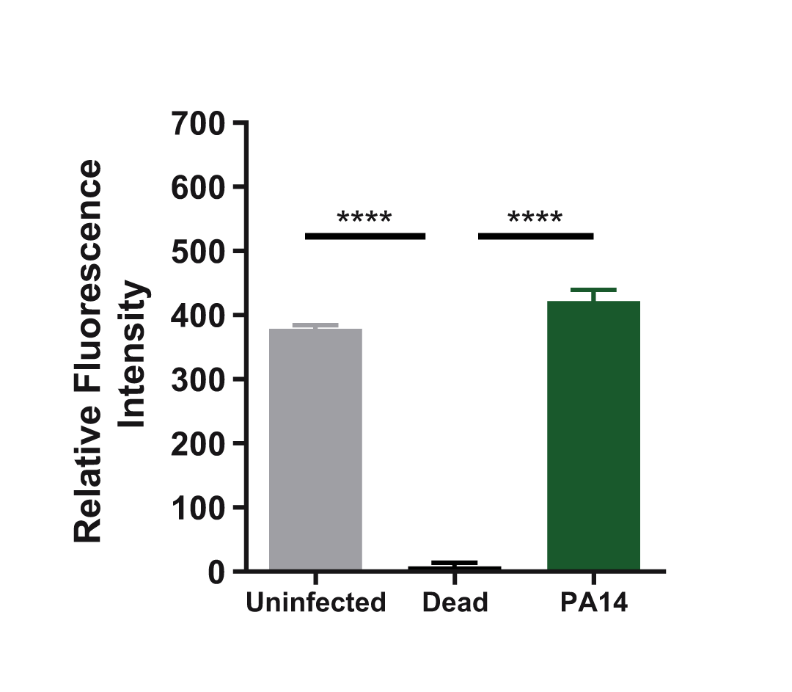
**

**Figure S1: Cell viability of epithelial cells one-hour post-infection with *P. aeruginosa***

Cell viability of uninfected, dead or *P. aeruginosa* PA14 infected A549 epithelial cells. Relative fluorescence intensity was calculated by subtracting the background fluorescence signal. Experiments were performed in independent triplicates for each condition. Samples were compared to negative control group using One-way ANOVA followed by Dunnet’s test for statistical analysis. Error bars indicate standard deviation. The asterisks show statistical significance: *P* ≤ 0.0001.


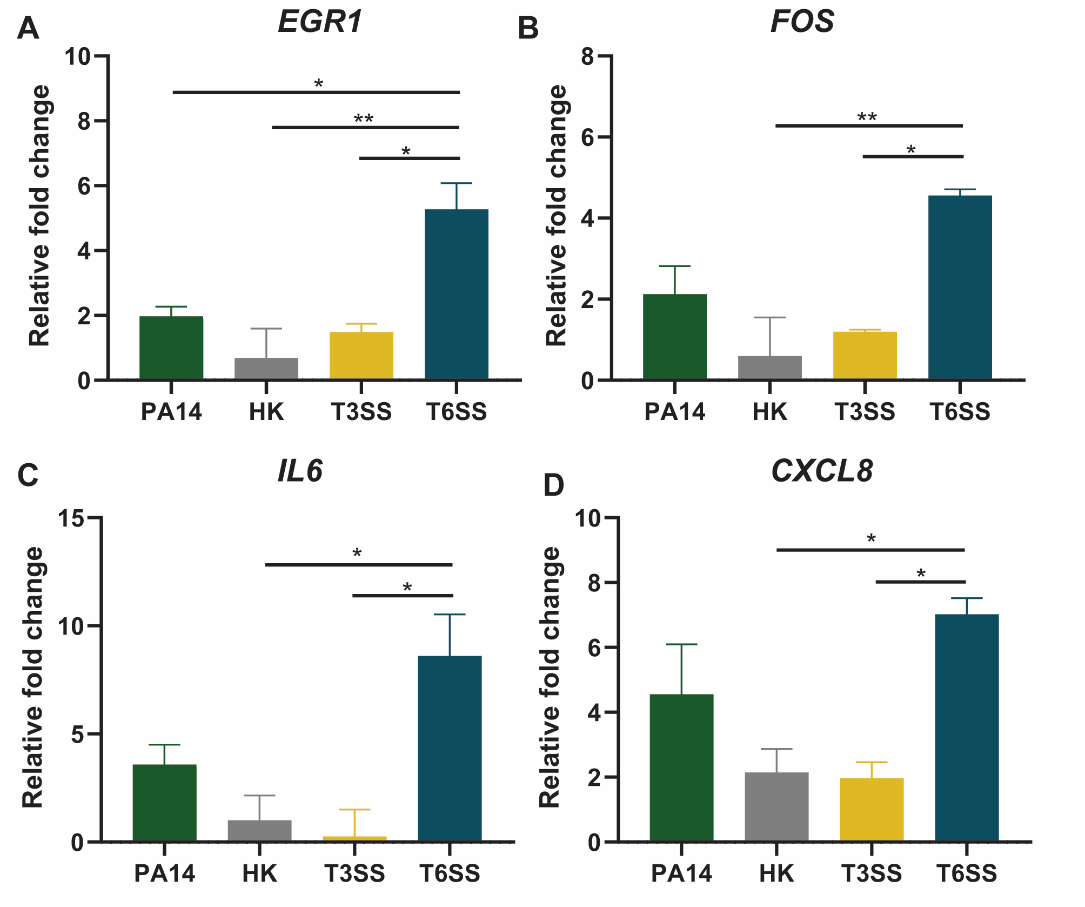


**Figure S2: qRT-PCR of selected genes in response to live and heat-killed PA14, and live T3SS and T6SS *P. aeruginosa* mutants**

qRT-PCR analysis of (A) EGR1, (B) FOS, (C) IL6, and (D) CXCL8 relative fold change compared to RPS13 housekeeping gene. Analysis was performed using three biological replicates with three technical replicates. Ordinary One-way ANOVA with Tukey’s multiple comparison tests was performed for statistical analysis (*, *p* < 0.05, **, *p* < 0.01)

**Table S1: Analyzed RNAseq data**

Each tab in the file lists the number of reads, RPKM, fold changes, *p-*values, annotations, and other relevant information for each comparison performed in this study.
